# Combined effects of *Ziziphora clinopodioides* essential oil and lysozyme to extend shelf life and control *Listeria monocytogenes* in Balkan‐style fresh sausage

**DOI:** 10.1002/fsn3.2141

**Published:** 2021-01-26

**Authors:** Maryam Ajourloo, Ali Khanjari, Ali Misaghi, Afshin Akhondzadeh Basti, Abolfazl Kamkar, Faezeh Yadegar, Fatemeh Gholami, Farzaneh Khansavar, Fazel Fallah

**Affiliations:** ^1^ Department of Food Hygiene and Quality Control, Faculty of Veterinary Medicine University of Tehran Tehran Iran; ^2^ Faculty of Veterinary Medicine University of Tehran Tehran Iran; ^3^ Research and Development Center Farsi Food Industrial Group Tehran Iran; ^4^ Research and Development Center Solico Meat Products Company Amol Iran

**Keywords:** foodborne pathogen, modified atmosphere packaging, natural antimicrobials, sausage

## Abstract

This study was done to evaluate the effects of different concentrations of *Ziziphora clinopodioides* essential oil (ZCEO) (0, 0.1, and 0.3%) and lysozyme (0 and 400 µg/g) on control of *Listeria* (L.) *monocytogenes* and also microbial, chemical, and organoleptic properties of Balkan type fresh sausage under modified atmosphere packaging (MAP) during 13‐day storage at refrigerated condition. Results revealed that treated sausages had a slower rate of increase in microbial count than control and sausages containing ZCEO (0.3%) and lysozyme (400 µg/g) possess the lowest microbial count at the end of the storage period. A reduction between 0.90 and 2.05 log CFU/g in *L. monocytogenes* was recorded for the treated sausage samples in comparison with control samples after 13 days of storage. Based on chemical findings, at the end of the storage, TVB‐*N* value in the control sample gradually increased to 34.30 mg/100 g, whereas TVB‐N values of the treated samples with each of the lysozyme and ZCEO alone or in combination were below 25 mg/100 g during the entire storage period. The final TBARS value for the control sample was 0.58 mg malondialdehyde/kg, while the TBARS values for the treated samples remained lower as 0.46 mg malondialdehyde/kg. Regarding sensory attributes, adding ZCEO results in insignificant lower scores in odor and taste than control in the early days of the study (*p* > .05). It can be argued that ZCEO alone or in combination with lysozyme showed good antimicrobial and antioxidant activities and may have this potential to be used as a preservative in fresh sausage without any significant adverse sensory effects (*p* > .05).

## Graphical abstract

Within this study, we evaluated the effects of different concentrations of *Ziziphora clinopodioides* essential oil (ZCEO) and lysozyme on control of *Listeria monocytogenes* and also microbial, chemical, and sensory properties of Balkan‐style fresh sausage under modified atmosphere packaging (MAP) during 13‐day storage at refrigerated condition.

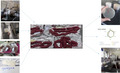

## 1 INTRODUCTION

Different kinds of sausages including cooked sausage, fermented sausage, and fresh sausage are produced and consumed all over the world. Using chemical additives and preservatives are usually avoided in fresh sausages. This type of sausage is highly perishable and has short shelf life. The short shelf life can be due to the high pH value (pH > 5.5) and water holding capacity (aw > 0.97) (Carballo et al., 2019; Cocolin et al., 2004). Balkan‐style fresh sausage is based on a simple mixture of beef and lamb meat, salt, pepper, garlic, water, and bicarbonate sodium, stuffed in natural casings (Carballo et al., 2019).

There are several published studies showing that *Listeria monocytogenes* was isolated from fresh sausage. The ability of this microorganism to grow at refrigerated condition can result in reaching its infective dose at low temperatures (De Cesare et al., 2007; Miyasaki et al., 2009; Rossi et al., 2011; Silva et al., 2004).

Using natural green preservatives such as essential oils and bacteriocins is a novel approach to extend the shelf life of fresh sausages and keep them safe (Carballo et al., 2019; Hugo & Hugo, 2015).

Essential oils (EOs) are classified as plant‐derived preservatives with the potential to decrease both pathogens and spoilage microorganisms growth (Khanjari et al., 2013). They are generally recognized as safe (GRAS) by the USFDA and regarded as safe food additives in the European Union at concentrations <2 mg/kg body weight/day (Commission, 2012; FDA, 2006).

*Ziziphora clinopodioides* is a species from the *Lamiaceae* family which is used traditionally as a flavoring agent in meat products in western regions of Iran (Shavisi et al., 2017). Many researchers have reported that extracted essential oil of this plant owns considerable antioxidant and antimicrobial activities (Gursoy et al., 2009; Ozturk & Ercisli, 2007; Shavisi et al., 2017).

Lysozyme, known as N‐acetylmuramide glycanhydrolase comprised of 129 amino acids, is an animal‐derived antimicrobial single peptide enzyme and considered GRAS by FDA (Chen et al., 2020; Syngai & Ahmed, 2019). It is documented that lysozyme has good antimicrobial activity, especially against gram‐positive bacteria. As it is a relatively heat‐stable enzyme, it can be applied to extend shelf life and to enhance the safety of a variety of foods (Aziz & Karboune, 2018; Janek et al., 2020; Pellegrini et al., 1992).

Using natural green preservatives and modified atmosphere packaging (MAP) simultaneously is a practical method to extend the shelf life of meat and meat products (Karabagias et al., 2011).

There are little, if any, published data on the use of *Ziziphora clinopodioides* essential oil (ZCEO) and lysozyme for enhancing the safety and shelf life of fresh sausages. This study was done in order to investigate (i) the effects of ZCEO and lysozyme for the control of *L. monocytogenes* inoculated (≃3 log CFU/g) in fresh sausage during a 13‐day storage period and (ii) the possibility of shelf life extension of fresh sausage using the above combination.

## 2 MATERIAL AND METHODS

### 2.1 Essential oil extraction and analysis

The fresh leaves of *Ziziphora clinopodioides* were gathered at the full flowering stage in May–July 2018 from natural growing plants located in Kermanshah Province Mountains, Iran. Then, ground air‐dried leaves (100 g) of *Z. clinopodioides* and distilled water (2000 ml) were subjected to a Clevenger apparatus system and steam distillated for 3 hr. Next, the collected essential oil was kept in an opaque glass tube under refrigerated condition until further use. The components of the used essential oil were identified by gas chromatography–mass spectrometer in our previous study (Hasan et al., 2019).

### 2.2 Balkan‐style sausage preparation

The preparation of sausage was done based on the method described by Carballo et al. (2019) which was modified in the following way. To prepare each treatment, 500 g beef and 500 g lamb meat was minced by a meat grinder machine equipped with a 5‐mm‐diameter sieve. Then, both minced types of meats were mixed together and sodium chloride salt (20 g) was added and mixed again for 10 min, and then, it was placed into a bowl covered with cling film and stored under refrigeration condition for 24 hr. On the same day, a mixture of 10 g finely cut fresh garlic and 1g pepper was boiled in 30 ml water for 2 min, then filtered, and cooled at refrigeration condition for 24 hr. At the following day, the minced meat was mixed with spice solution and 3 g sodium bicarbonate and different concentrations of ZCEO (0, 0.1, and 0.3%) and lysozyme (0 and 400 µg/g) for 10 min. After mixing, each batch was stuffed into lamb casings (inoculation for *L. monocytogenes* in selected batches was done before stuffing) and drained for 3 hr at 12°C. The sausages were divided into 100 g (for microbiological analysis) and 10 g portions (for *L. monocytogenes* growth analysis), which were individually packaged in bags (150‐µm‐thickness plastic film, having an oxygen permeability of 30 cm^3^/24 hr m^2^ bar at 23°C and 0% relative humidity) with modified atmosphere including 20% CO_2_ and 80% N_2_ and afterward stored at refrigeration condition (Carballo et al., 2019).

### 2.3 Inoculum preparation

*Listeria monocytogenes*, American Type Culture Collection (ATCC) 19117, was refreshed according to the method described by Khanjari et al. (2019) as follows: The lyophilized culture was transferred into tubes containing 10 ml of brain–heart infusion (BHI) broth medium and incubated twice for 18 hr at 35°C. Preparation of inoculation dose was performed by adjusting optical density (OD) (absorbance) of second BHI broth culture of *L. monocytogenes* to 0.1 at 600 nm long‐wave, using a Spectronic spectrophotometer (Jenway 6100, England). After that, decimal diluting of the resulting culture (OD 600 nm = 0.1) by 9 ml of 0.1% sterile peptone water was done to obtain viable cell counts of approximately 5 log CFU/ml. Finally, 100 µl of suspension was inoculated in ten‐point of sausage ingredient (10 g) (Khanjari et al., 2019).

### 2.4 Microbiological analysis

Sausage samples (10 *g*) were homogenized with 90 ml of 0.1% sterile solution of peptone water for 2 min, using a stomacher (Interscience) at 6 intervals during storage time (0, 2, 4, 6, 9, and 13 days). Afterward, appropriate decimal dilutions of each sample were prepared and cultivated in duplicate on the selected media and incubated, according to the procedure described by the culture media manufacturer, as follows: plate count agar (PCA) incubated at 30°C for 72 hr for the enumeration of total mesophilic bacteria and incubated at 7°C for 10 days for the enumeration of psychrotrophic bacteria; De Man, Rogosa and Sharpe agar (MRS agar) for lactic acid bacteria at 30°C for 72 hr; Violet red bile glucose agar (VRBGA) for *Enterobactriacea* at 37°C for 24 hr; and Palcam agar for *L. monocytogenes* at 30°C for 48 hr (Carballo et al., 2019; Esmaeili et al., 2020; Rezaeigolestani et al., 2017).

### 2.5 Chemical analysis

The thiobarbituric acid reactive substances (TBARS) according to the method described by Nam and Ahn (2003) were as follows: Fresh sausage samples were transferred to 50‐ml test tubes containing 15 ml deionized distilled water (DDW) and homogenized for 15 s. After that, 1 ml of homogenized samples was transferred to disposable test tubes (13 × 100 mm) and then butylated hydroxytoluene (7.2% in ethanol, v/v, 50 ml) and thiobarbituric acid/trichloroacetic acid (20 mM TBA/15% TCA, w/v, 2 ml) solutions were added. Next, the mixture for each sample was vortexed and placed in a 90°C water bath for 15 min to develop color. After cooling for 10 min in cold water, each sample was vortexed and centrifuged at 3,000 *g* for 15 min at 5°C. Finally, the absorbance of the resulting upper layer was read at 531 nm against a blank (1 ml DDW and 2 ml TBA/TCA solution). The amounts of TBARS were expressed as mg of malondialdehyde (MDA) per kg sausage (Nam & Ahn, 2003).

The total volatile basic nitrogen (TVB‐N) content of the sausage samples was assessed as previously described by Zhang et al. (2019) as follows: sausage sample (10 g) was transferred into Erlenmeyer flask, containing 100 ml distilled water, and was shook for 30 min, and then, the solution was centrifuged at 3,000 rpm for 10 min. After that, the solution was filtered through the filter paper, and 5 ml of filtrate was mixed with 5 ml of 10 g/L magnesium oxide. Steam distillation was performed using a Kjeldahl distillation unit for about 5 min. The distillate was absorbed by 10 ml of 20 g/L boric acid and then titrated with 0.01 mol/L HCl (Zhang et al., 2019).

### 2.6 Sensory evaluation

The fresh sausage was cooked by an electric grill in order to its core temperature reached 71°C. Random three‐digit blinded code samples (rectangular pieces approximately 2 cm × 1.5 cm with 1cm thickness were cut from the center of sausage samples) were evaluated by a 9‐member trained panel under similar location, light, and dish conditions. The order of sample presentation was randomized for each panelist and three replicates of all treatments were evaluated by them during the storage period. Palate cleansers, room temperature distilled water and unsalted crackers, were provided between samples (Araújo et al., 2018; Rezaeigolestani et al., 2017). Indices of color, odor, and taste were rated from 0 (corresponding to a least liked sample) to 5 (corresponding to the most liked sample).

### 2.7 Statistical analysis

The recorded data were analyzed using SPSS software (version 16.0 for Windows), and statistical significance (significance level was considered *p* < .05) among parameters in each day was evaluated by one‐way analysis of variance (ANOVA) and followed by Tukey test. Trends of changes in each treatment in different days were analyzed statistically using repeated measure test. After that, results were declared as mean value with their standard deviation (mean ± *SD*). The normality of the data was evaluated by one‐sample Kolmogorov–Smirnov test. All measurements were performed in triplicate (Khanjari et al., 2019).

## 3 RESULTS AND DISCUSSION

### 3.1 Chemical composition of ZCEO

The major constitutes of used ZCEO in the current study were geraniol (20.62%), carvacrol (18.17%), alpha‐terpineol (7.49%), 4‐terpineol (6.83%) and borneol (3.67%), and gamma‐terpinene (3.53%). The amount of carvacrol and thymol in used essential oil is in accordance with previous studies which showed that carvacrol and thymol are the major components of ZCEO (Aghajani et al., 2008; Karpiński, 2020; Shavisi et al., 2017). Nevertheless, pulegone was the major compound of ZCEO which was extracted in some studies (Amiri, 2009; HAYTA & BAGCI, 2016; Ozturk & Ercisli, 2007). The differences in the chemical composition of the ZCEO in various studies could be related to variation in age and growth phase of the plant, tilt the ground, altitude level, and geographical and climate conditions.

### 3.2 Microbial analysis

**FIGURE 1:**
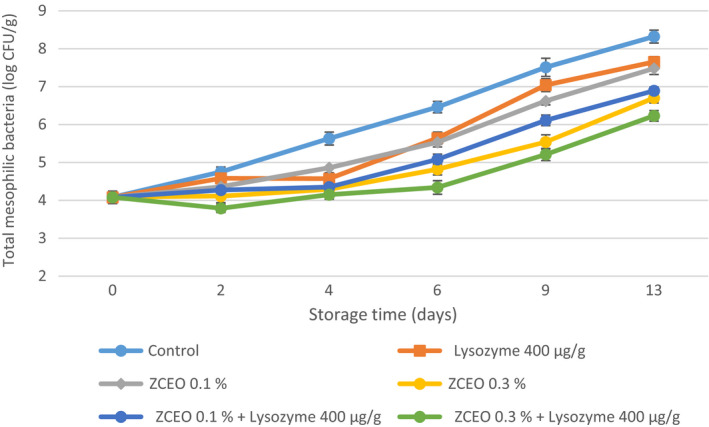
Changes in total mesophilic count (TMC) of fresh sausage under modified atmosphere packaging (MAP) during refrigerated storage (*Ziziphora clinopodioides* essential oil (ZCEO) and lysozyme)

Changes in total mesophilic counts (TMC) of fresh sausage samples during refrigerated storage under MAP condition are illustrated in Figure 1. The initial TMC of the fresh sausage sample was 4.08 log CFU/g and gradually increased to 8.32 log CFU/g at the end of storage. It was mentioned that TMCs of sampled fresh breakfast‐type sausages in Canada varied between 3.18 and 8.32 logs CFU/g with a mean of 5.65 log CFU/g depending on the formulation (Farber et al., 1988). Overall, there was a significant decrease in TMCs of sausage samples containing lysozyme alone comparing with control (*p* < .05), except for days 2 and 9. TMCs of the samples enriched with the ZCEO and lysozyme alone or in combination reduced significantly in comparison with control (*p* < .05). Also, adding ZCEO at a concentration of 0.3% alone or at concentrations of 0.1 and 0.3% combined with lysozyme into fresh sausage resulted in TMC below 7 log CFU/g until the thirteenth day. Similar results were reported by Da Silveira et al. (2014) who noted TMCs of fresh Tuscan sausage were significantly lower (*p* < .05) in the treated samples with 0.1% concentration of *Laurus nobilis* than in the control (da Silveira et al., 2014). With regard to the use of EOs in fresh sausage, the results of the current study are also in general agreement with those of Sojic et al. (2018), who reported that the addition of *Salvia officinalis* L. EO and *Salvia officinalis* L. extract significantly (*p* < .05) reduced TMC (Šojić et al., 2018). A similar finding was found by Zhang et al. (2019), who noted that TMCs were lower at 0.1% and 0.5% CinnamonEO treatment in comparison with control (Zhang et al., 2019). Gammariello et al. (2015) reported shelf life (according to TMC) about 18 days for fresh sausage by combining the dipping of meat first in sodium lactate solution (60%), then in optimal concentrations of EOs (1.25% fennel and 2.5% black pepper; 2.5% bay and 1.25% nutmeg), and packaging with MAP (30% CO_2_ and 70% N_2_) (Gammariello et al., 2015).

In a related study, Sharma et al. (2017) reported 1.65–2.72 log CFU/g reduction in TMC of fresh chicken sausages stored at 4°C for 10 days after the addition of clove EO (0.25%), holy basil EO (0.125%), cassia EO (0.25%), and thyme EO (0.125%) (Sharma et al., 2017).

**FIGURE 2:**
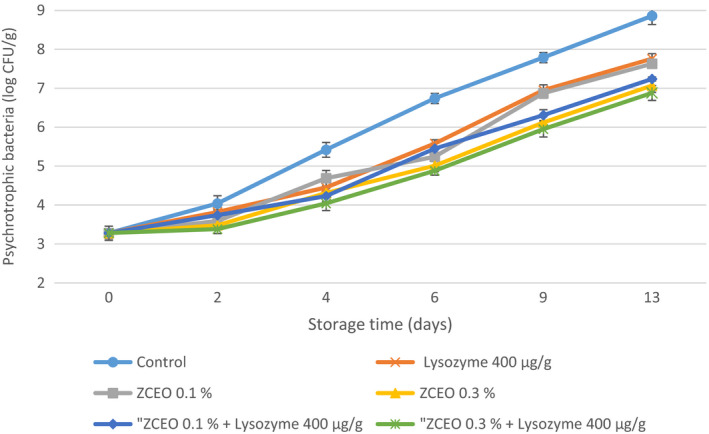
Changes in psychrotrophic bacteria (PSB) of fresh sausage under modified atmosphere packaging (MAP) during refrigerated storage (*Ziziphora clinopodioides* essential oil (ZCEO) and lysozyme)

The growth of psychrotrophic bacteria (PSB) under low temperature has a key role in reducing the shelf life of proteinaceous products (Shavisi et al., 2017). In the current study, the initial count of PSB in the fresh sausage was 3.28 log CFU/g and its final population decreased significantly (*p* < .05) (approximately 1.1–1.98 log CFU/g) in treated samples compared with the control sample (Figure 2). Nattress et al. (2001) evaluated the effect of lysozyme on two major spoilage psychrotrophic bacteria (*Brochotrix thermosphacta* B2 and *Carnobacterium* sp. 845), and their results revealed that lysozyme at a concentration of 500 μg/ml is able to inhibit *Brochotrix thermosphacta* B2 in pork juice whereas it showed no antimicrobial activity against *Carnobacterium* sp. 845 at concentrations of 1,000 μg/ml (Nattress et al., 2001). Therefore, it can be argued that lysozyme is capable to affect the growth of some psychrotrophic bacteria. Regarding the use of ZCEO, the results of the present study were in line with Sirocchi et al. (2017), who noted that the PSB count of beef decreased as 1.6 log CFU/g by the combination of MAP (50% O_2_ + 30% CO_2_ + 20% N_2_) plus *Rosmarinus officinalis* L. EO in comparison with control (Sirocchi et al., 2017). Da Silveira et al. (2014) also reported that PSB populations were significantly lower (*p* < .05) in the treated fresh sausage samples with bay leaf EO than in the control, except for days 0 and 4 (da Silveira et al., 2014). However, the contradictory finding was declared by Araújo et al. (2018), who reported that treating fresh sausage with a combination of garlic EO (0.0125 μl/ml), nisin (0.24 mg/ml), and allyl isothiocyanate (0.0125 μl/ml) had no significant effect on PSB population compared with control (Araújo et al., 2018). These differences can be related to the variation between the antimicrobial activity of various essential oils, the concentration of used essential oil, and initial PSB population.

**FIGURE 3:**
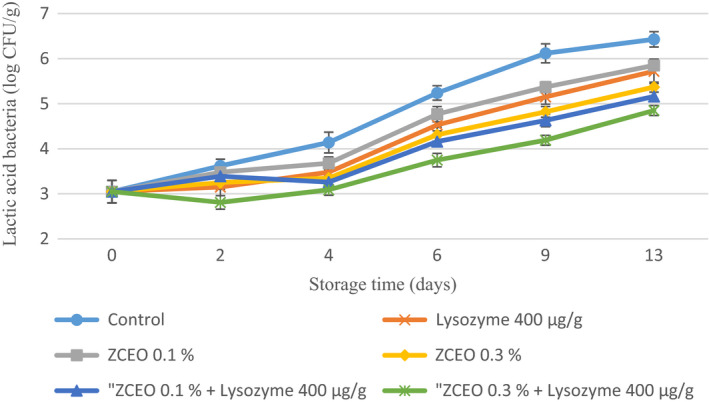
Changes in lactic acid bacteria (LAB) of fresh sausage under modified atmosphere packaging (MAP) during refrigerated storage (*Ziziphora clinopodioides* essential oil (ZCEO) and lysozyme)

It was documented that in vacuum and MAP condition, lactic acid bacteria (LAB) are among the most common microorganisms associated with spoilage of meat and fresh sausage (Carballo et al., 2019; Pothakos et al., 2015). Metabolizing of amino acids by LAB and producing organic acids, ammonia, and biogenic amines result in off*‐*flavor such as sour or putrid flavor in fresh sausage (Carballo et al., 2019). In our study, the initial count of LAB in fresh sausage sample was 3.05 log CFU/g and its final population decreased significantly (*p* < .05) in samples containing ZCEO alone or in combination with lysozyme compared with control sample (Figure 3). With regard to the use of MAP and antimicrobial compounds in fresh sausage, present results are in general agreement with those of Mastromatteo et al. (2011), who reported a significant reduction in LAB of Salsiccia, a type of fresh Italian sausage, after adding of thymol and the packaging with MAP (20% CO_2_, 5% O_2_, 75% N_2_) (Mastromatteo et al., 2011). Similar results were noted by Da Silveira et al. (2014) who found that adding bay leaf EO (0.05 and 0.1%) to fresh Tuscan sausage led to a significant decrease in LAB till day 6 of storage (da Silveira et al., 2014). Conversely, Carballo et al. (2019) reported that LAB count in fresh Balkan sausage treated with 0.1% *Zataria multiflora* EO did not show any significant decrease versus. control samples (Carballo et al., 2019). The possible reasons for the observed differences in LAB count can be attributed to the differences in used meat since in the current study the meat composed of equal content of beef and lamb meat whereas in Carballo et al. (2019) only lamb meat was used and higher amount of fat in lamb than beef results in protecting bacteria against EO (Perricone et al., 2015; Smith‐Palmer et al., 2001).

**FIGURE 4:**
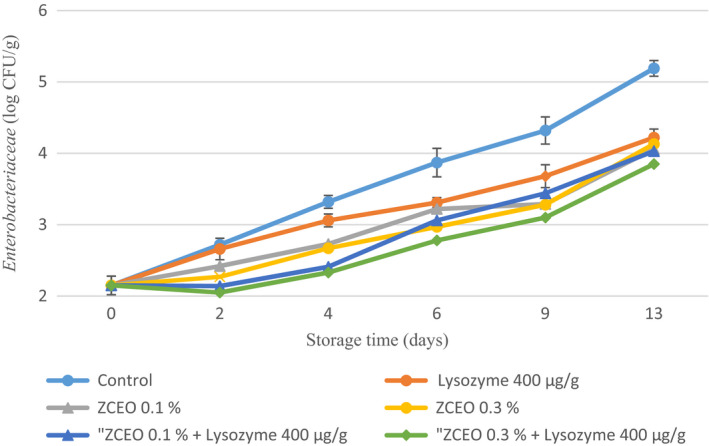
Changes in *Enterobacteriaceae* of fresh sausage under modified atmosphere packaging (MAP) during refrigerated storage (*Ziziphora clinopodioides* essential oil (ZCEO) and lysozyme)

The spoilage and health hazards related to the growth of *Enterobacteriaceae* in meat and meat products such as fresh sausages under MAP condition should be considered (Al‐Mutairi, 2011; Ercolini et al., 2006). Based on our findings, the initial *Enterobacteriaceae* count was 2.35 log CFU/g and reached 5.19 log CFU/g, and a significant (*p* < .05) reduction in the final population of *Enterobacteriaceae* by adding ZCEO and lysozyme alone or in combination was observed (Figure 4). Present results on *Enterobacteriaceae* evolution, regarding the use of essential oil, are in excellent agreement with those of Zhang et al. (2019) who mentioned that the addition of 0.1% or 0.5% cinnamon essential oil significantly decreased the counts of *Enterobacteriaceae* during 10‐day storage and also increasing EO concentration led to more reduction effect on *Enterobacteriaceae* count (Zhang et al., 2019). In the study carried out by Karabagias et al. (2011), it was illustrated that on day 9 of storage of lamb meat, *Enterobacteriaceae* reached 6 log CFU/g in control samples whereas in the presence of 0.1% thyme essential oil and MAP (80% CO_2_/20% N_2_), their population was reduced by 2.8 and 4.1 log CFU/g, respectively, as compared to controls (Karabagias et al., 2011). Del Nobile et al. (2009) declared that a significant decrease in *Enterobacteriaceae* count was observed in samples by the integration of thymol at concentration 500 and 750 ppm combined with MAP (40% O_2_, 15% CO_2_, and 45% N2) versus control samples in aerobic condition (Del Nobile et al., 2009).

**FIGURE 5:**
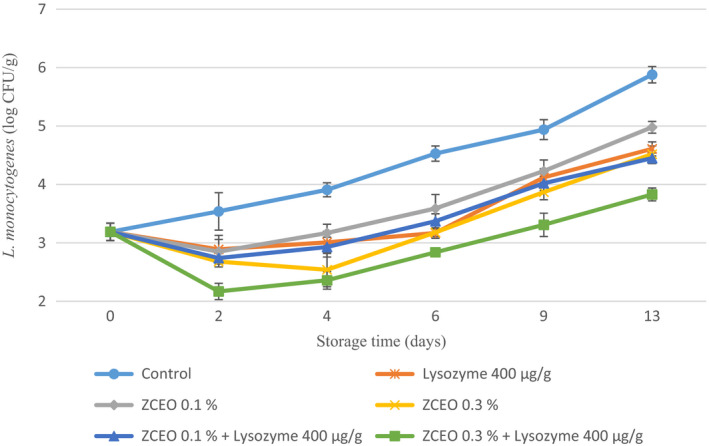
Changes in *Listeria monocytogenes* of fresh sausage under modified atmosphere packaging (MAP) during refrigerated storage (*Ziziphora clinopodioides* essential oil (ZCEO) and lysozyme)

The effect of ZCEO and lysozyme on the growth of *L. monocytogenes* in fresh sausage during storage time is given in Figure 5. It is shown that *L. monocytogenes* count in samples containing different concentrations of ZCEO and lysozyme was reduced significantly (0.9–2.05 log CFU/g) in comparison with the control after 13 days of storage. In a related study, Mastromatteo et al. (2010) reported a significant reduction in *L. monocytogenes* count by adding a combination of lysozyme (250 ppm), nisin (250 ppm), and disodium ethylenediaminetetraacetic acid (EDTA) (20 mM) in ostrich patties stored at 4°C for 8 days (Mastromatteo et al., 2010). However, contradictory findings were reported by Hughey et al. (1989) who found out adding 100 µg/ml of lysozyme to fresh sausages (bratwurst) inoculated with 3.60 log CFU/g of *L. monocytogenes* did not show any significant differences in comparison with control (Hughey et al., 1989). The possible reason for the observed difference in *L. monocytogenes* count with our study is due to the concentration of used lysozyme.

It can be argued that the reasons for the higher count of TMC, PSB, LAB, and *Enterobacteriaceae* in this study with regard to using MAP in comparison with some studies may be due to the procedure of preparation of Balkan‐style fresh sausage that minced meat was refrigerated for 24 hr after grinding and this can make an appropriate condition for microorganisms to grow. Besides, in the formulation of this sausage, adding 3% water leads to an increase in a_w_ of sausage. Furthermore, it was documented that MAP packaging composed of 20% CO_2_ and 80% N_2_ and absence of oxygen leads to an inhibitory effect on obligate aerobic microorganisms, as well as dissolving CO_2_ in water composition of meat and producing carbonic acid alters the interior pH of bacterial cells and suppress the growth of them. In the process of sausage production in the current study, bicarbonate sodium was added based on the recipe and this compound has buffering activity; thus, the intracellular pH does not change.

It is demonstrated that dominant constitutes of used ZCEO in this study are geraniol, carvacrol, and thymol that have antimicrobial activity. It was suggested that the antimicrobial activity of geraniol is related to its capability to penetrate into the interior of the bacterial cell and afterward interfering with intracellular sites and also inhibiting the efflux mechanism (Bhattamisra et al., 2018).

The possible mechanisms for antimicrobial activity of carvacrol and thymol could be attributed to structural and functional damages to the cell membrane by disruption of the outer membrane, interfering with membrane proteins and periplasmic enzymes (Di Pasqua et al., 2007; Helander et al., 1998; Jafri et al., 2019; La Storia et al., 2011; Moosavy et al., 2008).

It was documented that lysozyme is capable to hydrolyze peptidoglycan which is the main constituents of gram‐positive bacteria cell wall (Aziz & Karboune, 2018).

### 3.3 Chemical evolution

The mean protein and fat content of fresh sausages were 16.92% and 8.21%, respectively. It is proven that the TVB‐N index is a quantitative parameter that can be used to determine the freshness of meat and meat products (Zhang et al., 2019). The effect of ZCEO and lysozyme on the evolution of TVB‐N of fresh Balkan‐style sausage during the 13‐day storage at refrigerated temperature is shown in Table 1. As it is illustrated, TVB‐*N* values enhanced in both control and treated samples during storage time; however, the values were significantly lower in the treated samples in comparison with control (*p* < .05). The initial TVB‐N in the control sample was 10.21 mg/100 g which is in agreement with the previously reported results (Zhang et al., 2019), and after 9 days of storage, it reached to 27.49 mg/100 g and gradually increased to 34.30 mg/100 g at the end of storage. TVB‐N of the treated samples with each of the lysozyme and ZCEO alone or in combination were below 25 mg/100 g during the entire storage period. Our finding was comparably well in line with other researches focused on the impact of herbal essential oils in the reduction of TVB‐N in fresh sausage (Zhang et al., 2019). The lower TVB‐N values in treated samples may be due to the antibacterial activates of examined preservative agents in retarding bacterial growth followed by a decrease in the amount of nonprotein nitrogen compounds such as ammonia, primary, secondary, and tertiary amines (Shavisi et al., 2017).

**TABLE 1:** Changes in total volatile base nitrogen (TVB‐N) (mg/100g) of fresh sausage under modified atmosphere packaging (MAP) during refrigerated storage (*Ziziphora clinopodioides* essential oil (ZCEO) and Lysozyme)

	Sampling time					
	Day 0	Day 2	Day 4	Day 6	Day 9	Day 13
Control	10.21 ± 1.01^aA^	12.84 ± 1.29^aB^	17.38 ± 1.06^aC^	23.07 ± 2.14^aD^	27.49 ± 1.73^aE^	34.30 ± 1.17^aF^
Lysozyme 400 μg/g	10.21 ± 1.01^aA^	11.51 ± 1.18^aAB^	14.05 ± 1.23^abB^	16.95 ± 1.11^bC^	20.77 ± 2.14^bD^	23.81 ± 1.75^bE^
ZCEO 0.1%	10.21 ± 1.01^aA^	11.35 ± 1.10^aAB^	15.21 ± 1.41^abB^	17.60 ± 0.91^bC^	19.48 ± 1.52^bcD^	22.78 ± 2.24^bcE^
ZCEO 0.3%	10.21 ± 1.01^aA^	11.46 ± 1.27^aB^	13.20 ± 1.46^bBC^	15.42 ± 1.33 ^bCD^	18.22 ± 2.20^bcD^	20.65 ± 1.84^bcE^
ZCEO 0.1% + Lysozyme 400 μg/g	10.21 ± 1.01^aA^	11.66 ± 1.24^aB^	13.90 ± 1.38^abBC^	16.21 ± 1.77^bC^	17.13 ± 1.42^bcD^	21.56 ± 1.71^bcE^
ZCEO 0.3% + Lysozyme 400 μg/g	10.21 ± 1.01^aA^	11.61 ± 1.16^aB^	12.17 ± 1.19^bBC^	14.15 ± 1.32^bCD^	15.34 ± 1.93^cDE^	18.55 ± 2.21^cE^

Note

Different lower case superscript letters (a–c) show significant differences (*p* < .05) in the same column.

Different upper case superscript letters (A–F) show significant differences (*p* < .05) in the same row.

* The means of three replicates ± standard deviations are presented.

The second most known spoilage factor of fresh meat and fresh meat products is lipid oxidation or oxidative rancidity (Hugo & Hugo, 2015; Wenjiao et al., 2014). It is documented that factors such as light, oxygen presence, chemical characteristics of the meat, storage temperature, and technological processes affect lipid oxidation (Hugo & Hugo, 2015). Oxidation of fresh sausage lipids results in a decrease in its quality during the storage. The changes in TBARS values are shown in Table 2. The TBARS of the control treatment at the beginning of the study was 0.08 mg malondialdehyde/kg. In the control samples, the TBARS value reached to 0.58 mg malondialdehyde/kg, whereas the values for the treated samples remained lower as 0.46 mg malondialdehyde/kg. The results of this study revealed that under MAP condition during the entire storage period, the TBARS values of all samples did not exceed the reported threshold values for rancidity in meat products (Georgantelis et al., 2007; Verma & Sahoo, 2000). Present results, with regard to the use of ZCEO, are in general agreement with those of Sojic et al. (2018), who reported the antioxidative potential of *Salvia officinal* is L. EO and extract in fresh pork sausage, in which concentration of 0.1 μl/g of EO had the highest inhibitory potential against lipid oxidation (Šojić et al., 2018). The results of the current study were in line with the findings of Sharma et al. (2017) who noted that adding clove oil (0.25%), holy basil oil (0.125%), cassia oil (0.25%), and thyme oil (0.125%) to fresh chicken sausage led to a significant effect on TBARS values (Sharma et al., 2017).

**TABLE 2:** Changes in thiobarbituric acid reactive substances (TBARS) (mg malondialdehyde/kg) of fresh sausage under modified atmosphere packaging (MAP) during refrigerated storage (*Ziziphora clinopodioides* essential oil (ZCEO) and lysozyme)

	Sampling time					
	Day 0	Day 2	Day 4	Day 6	Day 9	Day 13
Control	0.08 ± 0.01^aA^	0.17 ± 0.03^aB^	0.27 ± 0.02^aC^	0.36 ± 0.03^aD^	0.44 ± 0.02^aE^	0.58 ± 0.02^aF^
Lysozyme 400 μg/g	0.08 ± 0.01^aA^	0.15 ± 0.02^abB^	0.24 ± 0.04^aBC^	0.31 ± 0.01^bD^	0.38 ± 0.02^bE^	0.46 ± 0.01^bF^
ZCEO 0.1%	0.08 ± 0.01^aA^	0.12 ± 0.01^abB^	0.18 ± 0.02^bBC^	0.24 ± 0.01^cD^	0.32 ± 0.02^cE^	0.41 ± 0.01^cF^
ZCEO 0.3%	0.08 ± 0.01^aA^	0.11 ± 0.02^bAB^	0.14 ± 0.02^bcBC^	0.21 ± 0.02^cdD^	0.26 ± 0.03^dE^	0.34 ± 0.02^deF^
ZCEO 0.1% + Lysozyme 400 μg/g	0.08 ± 0.01^aA^	0.09 ± 0.03^bAB^	0.16 ± 0.01^bcC^	0.22 ± 0.01^cdD^	0.29 ± 0.01^cdE^	0.37 ± 0.02^cdF^
ZCEO 0.3% + Lysozyme 400 μg/g	0.08 ± 0.01^aA^	0.10 ± 0.02^bAB^	0.12 ± 0.01^cC^	0.19 ± 0.01^dD^	0.25 ± 0.02^dE^	0.32 ± 0.01^eF^

Note

Different lower case superscript letters (a‐e) show significant differences (*p* < .05) in the same column.

Different upper case letters (A‐F) show significant differences (*p* < .05) in the same row.

* The means of three replicates ± standard deviations are presented.

In another study, Jridi et al. (2015) mentioned that using rosemary EO (500 ppm) results in a significant decrease in TBARS values in fresh sausage (Jridi et al., 2015). A similar result was reported by Zhang et al. (2019) who noticed that treating fresh sausage with 0.1 and 0.5% concentrations of cinnamon EO led to a significant reduction in TBARS values of treated samples compared with control (Zhang et al., 2019).

The antioxidant activity of EOs could be attributed to their radical scavenging nature, retarding growth of psychrophilic and mesophilic bacteria that are responsible for the oxidation of saturated fatty acids and chelating of metal ions (Bozin et al., 2007; Sharma et al., 2016; Sultzer, 1961).

### 3.4 Sensory evaluation

**FIGURE 6:**
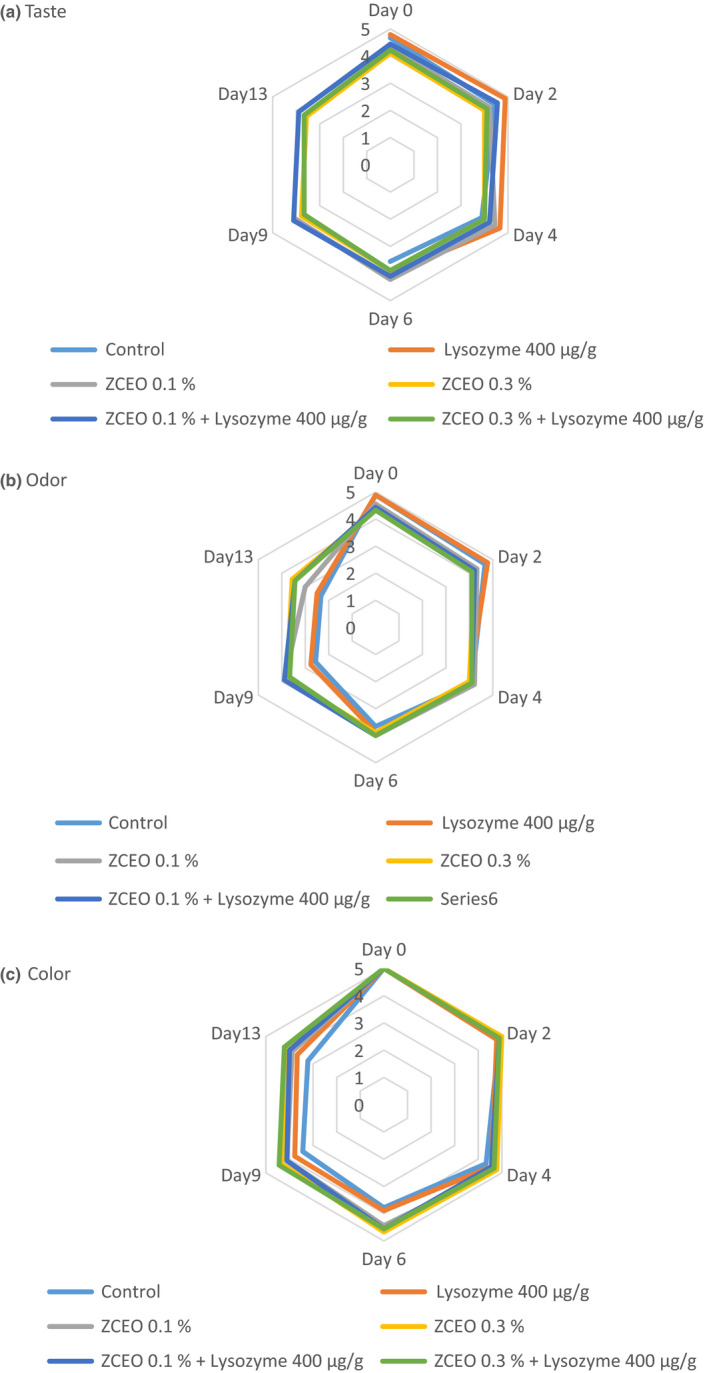
Effects of *Ziziphora clinopodioides* essential oil (ZCEO) and lysozyme on sensory attributes (taste, odor, and color) of fresh sausages under modified atmosphere packaging (MAP) during refrigerated storage

The results of the sensory analysis of fresh sausage samples are shown in Figure 6a–c. Regarding organoleptic attributes, adding ZCEO results in insignificant lower scores in odor and taste than control in the early days of study (*p* > .05). But this early negative influence of the ZCEO on taste and odor parameters of the treated sausages diminished during the storage period. Also, by increasing microbial and chemical values of sausage, the scores given by panelists to odor, taste, and color of samples were declined during the study period. The reason for this phenomenon can be attributed to the inhibitory activity of the ZCEO and lysozyme on microbial growth during refrigeration condition. The growth of *Enterobacteriaceae*, homofermentative LAB, and *Brochothrix thermosphacta* result in generation of some off‐flavor compounds such as acetoindiacetyl and 3‐methylbutanol. Also, LAB, *Enterobacteriaceae,* and *Brochotrix thermosphacta* are able to produce biogenic from enzymatic decarboxylation of free amino acids. These compounds lead to an unpleasant odor and taste. In addition, chemical compounds such as ketones and aldehydes resulted from lipid oxidation could be responsible for off‐flavors in stored sausages (Barbieri et al., 2019; Boylston et al., 2012; Carballo et al., 2019; Feiner, 2006; Shavisi et al., 2017).

## 4 CONCLUSION

Based on the findings of the current study, it can be concluded that using ZCEO at a concentration of 0.3% alone or at concentrations of 0.1 and 0.3% in combination with lysozyme successfully increased the shelf life of Balkan‐style fresh sausage under MAP during storage at the refrigerated condition. To summarize, these natural green preservatives may have this potential to be applied in fresh sausage without any adverse sensory properties.
